# Bit by Bit: Using Design-Based Research to Improve the Health Literacy of Adolescents

**DOI:** 10.2196/resprot.4058

**Published:** 2015-05-29

**Authors:** Mega Subramaniam, Beth St. Jean, Natalie Greene Taylor, Christie Kodama, Rebecca Follman, Dana Casciotti

**Affiliations:** ^1^University of MarylandCollege of Information StudiesCollege Park, MDUnited States; ^2^National Library of MedicineNational Institutes of HealthBethesda, MDUnited States

**Keywords:** health literacy, information literacy, computing literacy, consumer health, health informatics, K-12 education, adolescents, informal education, vulnerable populations, literacy programs

## Abstract

**Background:**

Although a low health literacy level has been found to be among the most powerful predictors of poor health outcomes, there is very little research focused on assessing and improving the health literacy skills of adolescents, particularly those from socioeconomically disadvantaged backgrounds. The vast majority of existing research focuses solely on reading comprehension, despite the fact that health literacy is actually a multifaceted concept, which entails many different types of skills.

**Objective:**

The aim of this paper is to first mine existing literature to identify the many different skills that have been posited to constitute health literacy, and then, using this collection of skills as an overarching structure, to highlight the challenges that disadvantaged youth participating in our HackHealth after-school program encounter as they identify and articulate their health-related information needs, search for health-related information online, assess the relevance and credibility of this information, and manage and make use of it.

**Methods:**

We utilized the design-based research method to design, implement, and revise our HackHealth program. To collect data regarding HackHealth participants’ health literacy skills and associated challenges, we used a variety of methods, including participant observation, surveys, interviews, focus groups, and logging of Web browser activities. We also collected data through specialized instructional activities and data collection forms that we developed for this purpose. Quantitative and qualitative techniques were used to analyze this data, as well as all of the artifacts that each student produced, including their final projects.

**Results:**

We identified the various challenges that the 30 HackHealth participants faced in completing various health-related information activities during the course of the program. Based on these findings, we describe important implications for working with youth from socioeconomically disadvantaged backgrounds, how to assess and improve their health literacy skills, and offer specific recommendations for health literacy instruction aimed at this population.

**Conclusions:**

With an increased societal focus on health and a shift from viewing patients as passive recipients of medical care to viewing them as active arbiters of their own health, today’s youth need to possess an array of health literacy skills to ensure that they can live long and healthy lives. Working with adolescents to help them develop and practice these skills will also help to break the cycle between poor health literacy and poor health outcomes, thereby reducing health disparities and improving the long-term outlook for the health of our nation.

## Introduction

### Background

A low level of health literacy has been found to be a stronger predictor of an individual’s health than age, race, education level, employment status, and income [1]. According to the National Assessment of Adult Literacy (NAAL), 36% of adults in the United States have limited health literacy (characterized by NAAL as basic or below basic health literacy), with lower health literacy levels even more prominent among immigrants, minorities, older adults, and lower-income populations [1,2].

But what exactly is health literacy? Due to the dynamic nature of health information, including its format, sources, and potential and actual uses, the definition of health literacy is evolving. Berkman et al [3] provide a complete retrospective and prospective view of health literacy that demonstrates the changes from individual-static definitions to individual-dynamic definitions to system-social definitions. What it means to be health literate has shifted from a narrow focus on one’s “ability to perform basic reading and numerical tasks required to function in the health care environment” [4] to a broader focus on possessing and applying a range of skills. According to NAAL, “Health literacy is not simply the ability to read. It requires a complex group of reading, listening, analytical, and decision-making skills, and the ability to apply these skills to health situations” [1]. Additionally, as the Internet becomes an increasingly integral part of the set of sources people regularly consult for information, we see novel challenges related to seeking, assessing, understanding, and using online health information.

According to Lenhart et al, 31% of teenage (aged 12 through 17) Internet users go online to find information about health, dieting, or physical fitness, and about 17% use the Internet to search for health topics that are difficult to discuss, such as drug use, depression, and sexual health. Further, teens from low-income households (< $30,000/year) are more likely than those from higher income households (> $75,000/year) to use the Internet to find health information (23% vs 11%, respectively) [5]. However, data regarding the health literacy levels of adolescents are not available [6], as this population has largely been ignored in health literacy research and intervention development [7,8,9,10]. Adolescence is a formative time when young people are in the process of developing health behaviors and habits that will influence their health in later years [6,9,11,12]. Thus, channeling energy and resources toward the development of health literacy and health information seeking skills among adolescents is vital.

In this study, we unpack the skills (which we term “health literacy bits”) needed to articulate one’s health-related information needs, search for health-related information online, assess the relevance and credibility of this information, and manage and make use of it. We draw from the various literatures that describe components of health literacy (ie, eHealth, information literacy, media literacy, computer literacy, digital literacy, etc). The primary objective of this study is to highlight the challenges that a group of disadvantaged adolescents participating in an after-school program (called HackHealth) face in acquiring these skills. We then provide recommendations for facilitating mastery of these skills among this population and share the changes we will make in the next iteration of the HackHealth program.

The HackHealth after-school program was designed to devise innovative ways to assist adolescents with health literacy development. HackHealth’s overarching goals are to increase adolescents’ interest in the health sciences, their health information literacy levels, their health-related self-efficacy, and their understanding of the crucial link between their daily health-related behaviors and their ability to maintain their health and prevent disease. Adolescents participating in the 8-week HackHealth program choose a health topic of personal interest, conduct research on this topic using both print and digital resources, create a final product incorporating what they have learned, and then present it to their peers and family members. Our curriculum (featured on the HackHealth website) focuses on topics such as keyword/query formulation, credibility assessment, topic refinement, and the use of online tools to create final products [13].

To date, the HackHealth program has been conducted in 3 Title I middle schools (ie, at least 40% of the school’s students are from low-income families [14]) in the mid-Atlantic region of the United States. All 3 schools have very high free and reduced-price meals program participation rates, ranging between 81% and 89% [15]. These indicators suggest that our research participants are from families of lower socioeconomic status. Middle schools in this region include grades 6 through 8, with the age of students falling between 10 and 15 years old. Each after-school session lasts between 60 to 120 minutes. Each school is staffed with a full-time librarian, all of whom participated in co-designing program activities using methods of cooperative inquiry [16-18] that utilized a variety of “low-tech” materials to create prototypes and sketches of HackHealth sessions. The librarians then implemented the HackHealth program in their school library alongside the research team.

### Prior Work

#### Adolescents and Their Information-Seeking Behavior

Health-related websites have become more prevalent and more diverse over the past 2 decades, growing to encompass newer types of content, such as user-generated content found in forums, blogs, and online communities. Adolescents are at an age where independent search becomes more enticing, particularly when their questions pertain to issues that are difficult to discuss [19-22]. Unfortunately, due to a relative lack of life experience and experience with searching [23], adolescents often have difficulty identifying relevant sources [24,25]. The various thoughts, feelings, and actions that typically occur during each stage of an individual’s search are identified in Kuhlthau’s [26] information search process model, clearly showing the challenges and resulting feelings youth experience.

A more tangible challenge to information seeking is the lack of basic access to computers. Without computer access, youth have limited opportunities to experience new open Web technologies, practice online searches, and gain experience with identifying the best search engines for their needs and the sources likely to contain the best information for their purposes [5,27]. Developing and refining questions is also difficult for youth, as they frequently lack domain knowledge [23,28,29] and may not understand the relevant terminology [30]. Their searching is frequently impeded by poor spelling ability [31], overreliance on website appearance when evaluating content [19,32-36], and a preference for search engines over library databases [37]. Even assuming that youth have access to computers and the ability to search for and evaluate health information, they may lack the motivation to do so. Youth may not conduct a search if they have no personal interest in a topic [38,39] or if they feel they already know the answer to a health-related question [40]. Similarly, they may not engage in a health-related information search due to a lack of self-efficacy in this area, as determined by associated factors such as optimism, persistence, and goal-orientedness [41,42].

Once youth locate health-related information, they need skills related to recall, numeracy, visual literacy, relevance assessment, and information integration in order to comprehend what they have found. Numeracy requires such basic knowledge as identifying numbers, but also requires computational, analytical, and statistical knowledge [43], much of which has yet to be taught to young adolescents. Youth often struggle with credibility assessment, automatically choosing search results that are listed first; believing websites in proportion to the amount of information they appear to contain; and/or using other novel, but often unreliable, methods [33,35,39,44]. Managing information requires skills in organization, such as storing information for future use. Individuals must analyze what information is needed and keep track of where they obtained it. However, this can be challenging, as adolescents are still “developing the cognitive ability to organize and logically process multiple information objects” [24].

Finally, once all of these steps have been completed, youth may then apply the information they have located by answering questions, solving problems, making decisions, advocating for others, and/or changing their behaviors based on what they learned during the search [1,4,45-52]. Youth may also communicate with others about what was found, which calls for an awareness of ethical issues, such as copyright infringement and privacy concerns. Such issues are incredibly complex and require abstract thinking, which is something adolescents are only starting to develop [24].

#### Health Literacy Skills

Due to constant changes in both the landscape of information and communication technology and in the personal, social, and environmental health needs of the individual, health literacy is not a static attribute of an individual, but rather a developmental process that unfolds over time [47,50,53]. Because health literacy is socially negotiated (ie, embedded within the social, cultural, and environmental context and norms of the individual and his/her community) [10,49,54-56]; contingent upon the resources an individual has access to at any given moment [54,56]; and variable dependent upon the complexity of the task at hand [56,57], we break down health literacy into individual skills, which we label here as “health literacy bits.” We use the term “literacy bits” to reflect the nature of the development of any type of literacy (ie, traditional, new media, health, etc), which is incremental and cumulative. Breaking the multifaceted construct of health literacy into separate literacy bits will enable health literacy teachers to focus on subsets of health literacy skills for a particular intervention.

We closely examined health literacy definitions and enumerations of associated health literacy skills (and literacies) from articles/resources that offered unique definitions and skills. (See our resulting health literacy skills inventory in Table 1.) We unpacked the skills and definitions that we found into 8 phases, with general abilities and characteristics (ie, health-related knowledge; ability to listen/communicate; motivation, attitudes, and intentions; and self-efficacy) and information access serving as the foundational elements/phases that must be in place in order to engage in the subsequent phases. In other words, without the motivation and self-efficacy to engage in health-related information seeking and use, and without information access, the other phases are likely to remain unattainable. Further, the extent and nature of an individual’s self-efficacy and information access will influence the fruitfulness of the other phases, which are information need identification and question formulation, information search, information comprehension, information assessment, information management, and information use. Although the use of a table structure and the term “phases” implies linearity, the health information seeking process is, in reality, a fluid and iterative process.

**Table 1:** Health literacy skills inventory.

Phase	Literacy Bit(s)	Source(s)
Foundational element: general abilities/characteristics		
	Health-related knowledge	48, 49, 50, 52, 57, 58, 59, 60
	Ability to listen/communicate	1, 11, 44, 45, 49, 50, 51, 54, 58, 59, 60, 61
	Motivation/attitudes/intentions	11, 45, 48, 52, 59, 61
	Self-efficacy	11, 47, 53, 59, 60
Foundational element: information access		
	Able to adapt to new technologies	46, 50, 51, 53, 61
	Aware of primary health resources to begin search	46, 47, 50, 51, 58, 62, 63
	Access valid information, products, and services	45, 47, 48, 50, 51
	Have exposure to computers in everyday life	46, 50, 53
	Awareness of search engines and their capabilities	50
Information need identification and question formulation		
	Develop and refine a range of questions to frame search	46, 50, 58, 64
	Understand relevant health terms	46, 47, 49, 50, 51, 57, 60, 63
Information search		
	Develop appropriate search strategies	46, 49, 50, 62, 64
	Use correct keyword searching strategies	65
	Use correct spelling in search terms	30, 65
	Use the library’s electronic resources, such as databases, etc	46, 64
	Maintain a critical stance, such as by using keywords that do not prematurely close off a search	11, 39, 64, 66
	Perform search informed by recommendations by health professionals and/or teachers (ie, reputed credibility)	68
	Understand how search engines work (ie, hits, order of search results, snippets, inclusion/placement of ads, etc).	46, 62
	Limit reliance on surface characteristics, such as the design of a website, the language used, etc (ie, surface credibility)	38
	Reduce search result selection based solely on word familiarity	38
	Use translation features on the search engine or Web page	38
Information comprehension		
	Able to read, comprehend, and recall information located	4, 11, 44, 45, 46, 47, 48, 49, 50, 51, 52, 53, 54, 57, 60, 61, 65
	Able to perform basic mathematical functions (ie, numeracy)	4, 11, 44, 46, 49, 50, 51, 52, 52, 53, 60, 61
	Able to comprehend simple charts (ie, visual literacy)	1, 46, 50, 61, 64
	Filter information found and extract only relevant information	46, 50, 58, 61, 62
Information assessment		
	Evaluate information based on its accuracy, validity, and appropriateness (ie, message credibility)	48, 50, 51, 53, 58, 61, 64, 69
	Evaluate source (eg, site sponsor or type of site (.com, .gov, .edu, .org)) to determine the believability of the person providing the information (ie, source credibility)	38, 53, 63, 68, 69, 70
	Evaluate the site based on when it was last updated (ie, currency)	64, 69
	Update generalized credibility perceptions, as applicable (ie, presumed and experienced credibility)	63
	Evaluate the credibility of the medium (ie, media credibility)	38, 45, 68
	Evaluate (not just accept without questioning) others’ claims regarding the validity of a site or of specific information (ie, reputed credibility, conferred credibility, tabulated credibility, emergent credibility)	38, 45, 46, 68
	Make sense of information gathered from diverse sources by identifying misconceptions, main and supporting ideas, conflicting information, point of view, and bias	28, 39, 53, 63
	Conclude which sites/information are valid and accurate by using conscious strategies (rather than simply using intuitive and heuristic judgments)	39, 50, 66, 67
	Refine search, as necessary	
Information management		
	Organize gathered information to optimize future retrieval/use	61, 64
Information use (dependent on context/goal of health information seeking)		
	Synthesize information from multiple sources; draw conclusions	50, 61, 64
	Answer questions originally formulated to represent information need	46, 50, 66
	Able to use information to address/solve health problems	4, 46, 47, 48, 49, 50, 51
	Use information located to make health-related decisions	1, 44, 45, 47, 48, 49, 50, 51
	Practice health-enhancing behaviors and mitigate/avoid health risks	45, 47, 48, 49, 51, 58
	Articulate potential limitations of published research findings and the cumulative impact of scientific knowledge (ie, incremental process of discovery) and wrong information	46, 53
	Share, collaborate, communicate, and create information, adapting as needed for intended audience (eg, self, peer, family, etc)	49, 50, 64
	Practice appropriate information ethics (eg, copyright, security, privacy, etc)	50, 64
	Advocate for personal, family, and/or community health	45

### Contribution of the Study

Through identification of the challenges encountered by HackHealth participants in the course of acquiring various health literacy skills, we will be able to ascertain the specific components of health literacy that we should emphasize in the next iteration of HackHealth. Additionally, we also examine our health literacy pedagogy (ie, our modules and our instructional strategies) in light of the challenges faced by HackHealth participants, and make targeted changes to the ways that we facilitate health literacy instruction. We also hope that our health literacy skills inventory and our revised HackHealth modules can serve as a guide to health educators who work with adolescents.

We will also use our health literacy skill inventory (Table 1) to develop a prototype digital health literacy assessment tool that will provide an objective measure of youths’ baseline health literacy level and their subsequent development of various health literacy skills, enabling us to move beyond a sole reliance on researcher observation and the few existing tools for assessing adolescents’ health literacy, which merely measure their perceptions of health information and of their own health literacy skills [6]. The inadequacy of existing health literacy assessment tools has been pointed out by many researchers [11,58-61]. In addition, there are few assessments of any type that focus specifically on the health literacy of adolescents [6,11,62,63]. Current instruments, such as the Rapid Estimate of Adult Literacy in Medicine-Teen (REALM-Teen), have primarily focused on reading ability and occasionally numeracy, rarely acknowledging the wider range of abilities and dispositions needed to achieve robust health literacy [58,64]. Considering the relative lack of knowledge about adolescent health literacy [9,10], the development of an appropriate tool to measure a comprehensive array of the skills involved in health literacy is critical.

### Objective of the Study

The objective of this study is to identify the challenges that a group of disadvantaged adolescents participating in the HackHealth program encounter in mastering the health literacy bits that we have identified from existing literature (Table 1). Using these literacy bits as an organizational framework, we provide salient examples of the common challenges that our participants faced as they navigated the various phases of health information seeking and use. Based on this analysis, we provide pedagogical recommendations that we will implement during the next iteration of our HackHealth program and that can be used by other health literacy coaches and teachers in their health literacy instruction.

## Methods

### Recruitment

Adolescents are recruited for the HackHealth program by school librarians through school-wide announcements, consultations with health teachers, and referrals by homeroom teachers. A total of 30 students across 3 schools have participated in HackHealth*,* comprising 7 (23%) boys and 23 (77%) girls. Four (13%) of our participants were in sixth grade, 14 (47%) were in seventh grade, and 12 (40%) were in eighth grade. Of these 30 participants, 20 completed the entire program. The range of ages of participants was 10 to 15 years old, averaging 12.8 (SD = 1.15). The vast majority were 13 (n=14; 47%) or 14 (n=7; 23%) years old. All 30 participants belong to minority groups in the United States: 15 (50%) are Hispanic/Latino, 10 (33.3%) are African American, 4 (13.3%) self-identify as “Other,” and 1 (3.3%) is Asian. A majority (n=22; 73%) of our participants reported that they have a computer at home, and most (n=21; 70%) had accessed the Internet from home using at least 1 type of device, such as their own or a parent’s computer, cell phone, and/or tablet.

### Data collection

Our approach is informed by design-based research (DBR), which is often used in the learning sciences [65]. DBR entails researchers and educators actively collaborating in designing and implementing learning programs and relevant technologies. Using both qualitative and quantitative methods, the researchers seek to collect rich data that uncovers the pedagogical and technological factors that play a role in students’ learning processes. This type of research is termed “design-based,” as data collection and the insights gained are continuously used to inform the subsequent design of the learning program and technology. The DBR process is iterative, and our goal is to continuously refine and develop the HackHealth health literacy curriculum.

Prior to commencing the HackHealth study, we obtained approval to conduct this study from the University of Maryland’s Institutional Review Board and the school district’s Department of Research and Evaluation. We held an introductory meeting at each school to explain the program to interested adolescents and to distribute parental and student consent forms. At the meeting, we explained the goals of the project, walked the students through the content on the consent forms that they and their parents would need to sign before they could participate, and addressed any questions they had.

To obtain a complete description of the health literacy perceptions and practices of our participants, we employed several different data collection methods throughout the 8-week program. Table 2 lists the data collection methods we used, along with the approximate time we spent implementing each of these methods.

Our data collection efforts resulted in a total of 650 pages of materials, 176 pages of observation notes, and 80 hours of audio recordings of our interactions with participants (which includes recording of interviews, focus groups, and 1-to-1 sessions). Interviews and focus group sessions were transcribed in their entirety, and other audio-recorded materials were transcribed as needed.

**Table 2:** Data collection methods.

Instrument	Session(s)	Approximate Time Spent	Description
Pre-/post-program survey	1 and 8	15 min	Collects participant demographics, preferred sources of health information, interest in science and health, and students’ perceptions regarding their health literacy skills
Topic and goal selection form	2	20 min	Collects students’ choice of topic, as well as their motivation for choosing this particular topic
Credibility screenshot activity	4	30-40 min	Using large poster-sized screenshots of 6 obesity-related websites—including a government site, a KidsHealth site, a blog, a Wikipedia site, a WebMD site, and a Dr. Oz site—we ask the students to place green sticky notes on the posters next to aspects of each site that they feel make the site credible and to put pink sticky notes next to aspects that they feel make the site not credible. Students write explanations on each sticky note. (For a more detailed description of this activity, see Subramaniam et al, 2015.)
Google search results activity	5	20-30 min	Using a printout of a Google Search results page for the keyword “obesity,” we ask students to put a star next to the 3 links they would most likely click on. We then engage them in a group discussion on the reasons for their choices. (For a more detailed description of this activity, see Subramaniam et al, 2015.)
Search log	4-6	30 min per session	Students fill out a search log form as they search for information regarding their selected topic. The form elicits the keywords they used for each of their searches; the URLs of the sites they visited; and their perceptions regarding the usefulness, credibility, and ease-of-use of each of these sites.
Final project goal sheet	4-6	5 to 10 min per session	The students fill out (and update, as needed) a form indicating their selected topic, the mode they will use to deliver their final project, and a list of the information and skills that they still need to complete their final project.
Participant observation	All	Eight 60- to 120-min sessions per school	All researchers attending the sessions conducted participant observation for the full duration of every session at each school.
Browser history downloading and documentation	Most sessions	30 min per session	Following the sessions during which students conducted research on their health topics, we collected their browser histories for future analysis.
Artifacts	Most sessions	Varies	This includes the research organizers (in which students recorded notes regarding what they were learning and the sources they consulted) and their final projects.
Post-program interviews	8	30 min per student	Interviews were conducted using open-ended questions that elicited students’ perceptions regarding the impacts of the program in terms of their interest in health, their learning, their health-related self-efficacy, etc.
Focus group	Final party/focus group	60 min	The questions focused on students’ experiences during the program. Focus group size is between 3 to 8 students.

### Data Analysis

Two members of the research team undertook the data analysis. They began by practicing open coding on a complete data set from 1 participant and all researcher observation notes from 1 week at 1 school. Each researcher developed a personal codebook based on the research goals articulated for the study and the health literacy skills inventory (Table 1). By comparing their personal codebooks, the researchers identified the central themes of interest, resulting in the development of a shared codebook. The final version of the codebook was entered into NVivo 10 qualitative data analysis software and then used to code the remaining data. Each data artifact was coded by 1 researcher, with another researcher checking the codes for agreement. As additional codes emerged during the coding process, the researchers discussed how to categorize and define them. Where there were disagreements, the excerpts were discussed. Extensive memos were kept of coding decisions to establish an audit trail.

## Results

### Overview

Based on analysis of the data we collected, we highlight the challenges that participants encountered in their health information-seeking, as these challenges reflect the deficits in their health information literacy. Our analysis is structured by the literacy bits outlined in Table 1, focusing on the literacy bits that are delineated in each of the information-seeking phases. As the 2 types of foundational elements—general abilities/characteristics and information access—delineated in the first 2 sections of the table are required for a student to be able to engage in information-seeking phases, we focus this paper only on the literacy bits that pertain to participants’ actual information need identification/articulation, information seeking, information comprehension, information assessment, information management, and information use processes. For each bit, we share relevant quotes, observations, and/or narrative that exemplify the nature of these challenges. In this paper, we utilize the pseudonyms personally selected by participants. Our analysis is based on data collected throughout the program, but we only highlight the literacy bits that participants had trouble with, excluding those that adolescents appeared to have mastered or that we did not have a chance to observe.

### Information Need Identification and Question Formulation

#### Difficulty Understanding Relevant Health Terms

Although our participants were quite accomplished at formulating research questions, they sometimes lacked familiarity with relevant health terminology, which affected their ability to frame and/or refine their questions and to understand and make use of the information they located. Many participants conducted 1 or more definition searches embedded within their information searching processes. For example, 1 researcher described the following regarding her observations of Betty Boop’s information searching processes: “She selected a link to a MedlinePlus article called ‘Infant of diabetic mother.’ She found this article extremely useful; however, it contained several vocabulary words that she was not familiar with, such as ‘bilirubin’ and ‘neonatal polycythemia.’ . . . When she came to words that she didn’t know . . . she would open another tab in the browser and type in the URL bar ‘definition of [fill in the blank].’ For example, she looked up ‘bilirubin’ this way, but said that the definition provided wasn’t useful because it used the word ‘hemoglobin,’ which she also didn’t know. At 1 point, she ended up with multiple tabs open, looking up unfamiliar words contained in the definition of the words she had originally looked up.” [Observation 01/29/2014] This often-multitiered chase after the meanings of unfamiliar health terms frustrated our participants and sometimes caused them to lose focus of their original questions.

#### Difficulty Framing Relevant Search Queries

Another challenge they faced was a lack of familiarity with health terms to use in their initial search queries in order to retrieve accurate, relevant information. For example, Emily was searching for information on “cancer in blood” and was unfamiliar with the term “leukemia,” until a researcher shared it with her. A researcher observed, “It is fascinating how the framing of a search [for] ‘cancer in blood’ versus ‘leukemia’ makes a difference [in the search results].” [Observation 08/10/2013]

### Information Search

#### Use of Less Effective Keyword-Searching Strategies

Many HackHealth participants used 1-word queries to search for information. We observed some of them typing 1-word queries, such as Captain searching for “concussions” and Jerry looking up “coma,” which returned so many results that participants spent a lot of time just sifting through them. As 1-word searches are typed into search box, participants frequently use Google’s “autocomplete” feature that offers suggestions as a searcher is typing his/her query. Our observations revealed that participants typically use the autocomplete feature for 1 of the following reasons: (1) they know the broader topic that they are interested in, but don’t know the specific subtopic, questions, or proper search terms that should be used; (2) they know the search terms that they would like to use, but don’t know the exact spelling for the terms; or (3) they do not know the actual terminology for the illness or health topic that they are interested in and rely on autocomplete for suggestions. For example, Jerry knew he was interested in researching comas, but he relied on autocomplete to select his particular subtopic of interest. We found that our participants frequently clicked on 1 of the search suggestions brought up by the autocomplete function, even if it was not what they had intended to type in the search box in the first place.

#### Lack of Understanding Regarding How Search Engines Work

Our participants had little understanding of the organization of the search results page. They did not always distinguish between ads and other search results. For example, they paid little attention to the presence of links to advertisements that appeared among their search results, and at times, believed that the advertisements, which were listed at the top of the search results page, were actually credible sites to obtain health information. For example, 1 researcher observed, “Ariana then went to Google and typed the query ‘how to cure heart disease.’ She clicked on the first ad, which took her to the Mikey Network... ” [Observation 01/14/2014] Participants did not understand the order of the search results page, believing the list to be alphabetical or based on popularity. Andy Sixx, for example, believed that search results were returned alphabetically. Jerry believed that the top search results (starting with the ads) are the most popular. Some participants relied solely on the snippets, rather than clicking through to the website, as 1 researcher reported, “When [Ariana] got the search results for each of these searches, she just used the snippets to get the information needed and . . . did not actually click any of the links.” [Observation 02/26/2014]

#### Tendency to Select Search Results Based on Word Familiarity

We observed an overreliance on word familiarity among many of the participants who come from non-English speaking homes. During 1 of the activities designed to capture how they decide which search results to click on, we discovered that they were relying on recognizing words that were familiar to them. For example, 1 student chose a link entitled “Obesity Information” just because she recognized the term “information,” even though the listing was an advertisement on the search result page. Once arriving on a page, another student deemed it trustworthy because it had the option to translate the page to Spanish. More information on this phenomenon can be found in Subramaniam et al, 2015.

### Information Comprehension

#### Difficulty Reading, Comprehending, and Recalling Information

The primary obstacles that hinder these adolescents from comprehending the health information they find on the open Web are a lack of mastery of the English language and an inability to understand the health terms/phrases that are embedded within the information they find. In addition to unfamiliarity with scientific or health-related terms, participants typically do not recognize common health terms such as “symptoms” and “diagnosis.” For example, a researcher noted, “Nicole’s vocabulary seems to be a barrier . . . I had to explain what the terms ‘symptoms’ and ‘diagnose’ meant.” [Observation 11/12/2013]

#### Trouble With Filtering Information Found and Extracting Only Relevant Information

While we did have participants who actually read through paragraphs of texts from their selected information sources with tremendous patience and attempted to capture only the relevant information, the majority of participants skimmed through Web pages rather quickly and sometimes missed relevant information and/or ended up with the wrong information. For example, 1 researcher wrote, “[Mr. Science Guy] was skimming too quickly to retrieve good information. In point, he ended up with completely incorrect information, though the site has it spelled out correctly. . . . [Similarly, Star Wars] read off the article’s main points, though like Mr. Science Guy she ended up with misinformation because she perused much too quickly.” [Observation 05/01/2014]

### Information Assessment

#### Difficulty With Evaluating a Source to Determine Its Credibility

One common reason participants trusted information was because the author had professional expertise, such as in the case of a doctor, but they also extended their perceptions of credibility to news reporters and celebrity doctors. Jaysa explained her belief in reporters: “They are reliable, the news reporters, ’cause, you know, I don’t think they would lie.” Nunu identified Kathleen Doheny as the author of a WebMD article that she had found, and learned that she’s a journalist who specializes in health, fitness, and behavior topics. Nunu explained why she trusts her: “She’s a journalist, but . . . she still knows about health topics and fitness and stuff. Even though you’re not a doctor, you might still know a little about the health-related topic.” Betty Boop said that she trusts the Dr. Oz website because her mother watches his show and has used some of the information he provided and found that it worked.

At times, our participants faced challenges in correctly identifying the true source of information on the Internet. Sometimes they could not find an author name. Other times, they incorrectly inferred who the author was. For example, several students believed that WebMD is written by doctors from Maryland. Captain said, “The first website I would go to is WebMD because . . . it’s doctors from Maryland.” Another student, Phenomenal, incorrectly identified the webmaster of a site as the author of the content on the site.

Participants also encountered problems as they evaluated the credibility of a website based on its domain. They tended to feel that .org websites were more trustworthy than .com sites. Chocolate Rain, for example, explained, “It’s a .org website, so you know they’re not getting too much money from it, I guess. . . . Most .com websites, even though some of them have good information, some of them . . . get money from it.” However, participants sometimes found it confusing to try to judge a site based on its domain. As Chocolate Rain put it: “I’m still confused because it’s like, how do we trust the sites, because . . . most of us trust .org and then some of us don’t trust .com because most of .com’s are being paid by . . . they’re like money sites. And then we don’t trust .gov because it’s the government, but then there’s Wikipedia.org. I’m just like, . . . it’s a .org site, why don’t you trust it?”

#### Difficulty Evaluating the Credibility of the Medium

Sometimes participants felt that information on the Internet was only credible if it retained features from generally trusted media, such as books. Cherry Marshmallow stated, “I found out from my friends, if it [the website] has, you know, those copyright things in a book, if it has that, it’s credible . . . that’s what they say.” Some participants also felt that online information was credible if they already knew the source from another context. Little Man said he trusted the Let’s Move website because he had seen Mrs. Obama talking about this website in a television ad. Star Wars agreed that this website is credible, explaining “because Mrs. Obama said so.” However, there is also danger of completely trusting everything on the open Web, as Ariana explained, “It has to be true because you can’t put fake stuff on the Internet!”

For some participants, the presence of special types of content or functionality—such as pictures, videos, or the ability to listen to the content read aloud, to read the content in Spanish, or to perform a search on the site—signaled credibility. Chocolate Rain, for example, explained that a particular website was credible because she had used it previously and found that “it has extra things, like you can listen to it . . . so if you have trouble reading, it reads to you, and then it has it in Spanish and . . . different things.” However, other types of functionality, such as the ability to log in (which is intended for users to save searches within WebMD, among others), signaled a lack of credibility to some participants. Jaysa, for example, felt that WebMD is not credible “because you can see right here, sign up . . . or sign in, and then it’s like a blog, so no.”

#### Difficulty Evaluating Others’ Claims Regarding the Validity of a Site or of Specific Information

Some participants described trusting (or distrusting) a site because a relative or teacher trusts (or distrusts) it. Cherry Marshmallow, for example, said she trusts WebMD because her mom trusts it. Mr. Science Guy stated, “I know I went there (teenshealth.org) ’cause my teacher said it was a reliable website.”

A few participants used social measures, such as number of followers or number of viewers, as indicators of credibility. Jerry, for example, explained that he chose the link DonorsChoose.org because he noticed that it had 2,609 followers on Google+. Sometimes, however, they misread these cues. For example, Phenomenal said that a diabetes-related video he watched was credible because the person who posted the video had posted many other videos on this topic. However, the figure he was referring to actually reflected the number of Internet users who had viewed this particular video.

#### Use of Inaccurate Strategies for Cross-Checking Information From Multiple Sources

Out of the 30 HackHealth participants, only 5 deliberately compared (or mentioned that they will compare) the information found across multiple sources/websites to verify or triangulate information. For example, Chocolate Rain mentioned in her post-program interview that she will “check and see . . . other sites have the same information” before actually deciding to use the information that she found. However, even these 5 participants do not exercise credibility assessment strategies in parallel with the synthesis of information from multiple sources. For example, as 1 researcher reflected on JMoney’s strategy, “ . . . [S]he compared the information she found at several sites and if a lot said the same thing she knew it was right. She didn’t really mention the 5Ws,” which is the credibility assessment checklist that we provided to the participants. [Observation 05/01/2014] Another example is Jerry, who always clicks on the Wikipedia link first, and said to just “search another website to just double-check if it’s right . . . ,” which indicates his eagerness to validate the information in Wikipedia by comparing it with other sites. Some participants (Jerry and Jaysa) say that they will check “another” site and some (Kaylee, Chocolate Rain, and JMoney) mentioned that they will compare the information found with “several” and “other” sites, referring to more than 1 other source.

### Information Management: Difficulty Organizing Gathered Information to Optimize Future Retrieval/use

Participants do not generally organize the information they find so as to make it easy to return to for use in their presentations. A researcher observed that Nuya “skimmed [websites] hastily and took notes haphazardly . . . currently, her results and notes are mixed for all types of cancer.” [Observation 01/15/2014] Some participants made no (or very few) notes after identifying information that could be used for their presentations. For example, Nunu had no notes at the point when she wanted to create her presentation and primarily worked from her memory of the information that she read.

### Information Use: Unawareness of Appropriate Information Ethics

We did not have many opportunities to observe participants’ information ethics practices in using the information they found, with the exception of their citation/referencing practices and note-taking strategies. Participants generally did 1 of the following: (1) used information without citing because they are ignorant about the need to do so; (2) forgot to cite the resources that they used but know that they should have; and (3) did not know how to cite the sources that they used. Regarding (1), 1 researcher wrote, “Arianna had heard about the word plagiarism but she didn’t know what it meant.” [Observation 02/19/2014] Regarding (2), the HackHealth team noticed many instances where participants failed to cite the sources that they used in their presentations. When this point was brought up with them, participants often readily agreed that they should have done so. Regarding (3), participants were frequently unsure of when to cite and how to go about including references in their slides. For example, when Andy Sixx noticed that some of her peers had references in their presentations, she checked with a researcher to find out how to include the references she had used in preparing her slides.

Participants often lacked ethical note-taking strategies. A common observation among researchers is the tendency for participants to copy word-for-word the information they found. For example, as a researcher observed, Betty Boop “took extensive notes, pretty much copying word-for-word what was on the Web. . . .” [Observation 01/29/2014] The same researcher observed another student, “Arianna really wanted to just copy-paste text from the Internet directly into her presentation. She explained that she didn’t like having to switch windows back and forth in order to be able to type things in her own words.” [Observation 02/19/2014]

## Discussion

### Principal Findings

Through our experiences working with adolescents in the HackHealth program, we have identified several challenges that they encounter when moving through the phases involved in identifying an information need, formulating one (or more) specific questions, looking for information, and processing, assessing, managing, and using online health information. In this section, we identify some of the implications of our findings, describing the types of learning activities that we have specifically designed in order to try to address these particular areas of difficulty. Next, we outline the limitations of our work and the novel contributions that we are making to both practice and research related to health literacy instruction for this population. In conclusion, we discuss the importance of increasing young people’s motivation, self-efficacy, and health literacy to enable them to live long and healthy lives, as well as the role of HackHealth in carrying out this important mission, thereby helping to reduce health disparities and improve the long-term health outlooks for participating youth.

### Implications

Findings from our work with HackHealth participants raise a number of important implications for working with youth from socioeconomically disadvantaged backgrounds to assess and improve their health literacy skills. First, we need to ensure that they have the basic necessities, as laid out in the foundational elements sections of Table 1. That is, they need to have a basic level of health-related knowledge and an ability to effectively listen and communicate, as well as the motivation and self-efficacy needed to engage in health-related information need identification and information seeking and use. They also need to have access to information and information technologies, an awareness of some trustworthy sources of health information, and a basic understanding of how search engines work.

Moving beyond the foundational elements, today’s youth need to have a toolkit of search strategies to use if their preferred method proves unsuccessful. They could use help with converting an information need into a question and then converting a question into a search query. They also need to understand how Google (their preferred search engine) works—for example, how the order of search results is determined—and that Google’s autocomplete feature can be, but isn’t always, helpful. Importantly, they need to have lots of opportunities to practice searching, as well as adequate instruction in this area. Ideally, this will help to make them more comfortable with online searching, thereby reducing their anxiety and frustration. Based on these needs, we have revised our original HackHealth modules (we call them “pods”) to include more streamlined instruction on research question and query formulation in addition to activities that focus specifically on how the Google search engine selects returns based on a user’s search terms/query. For example, one of our activities details the various aspects of a search engine results page and how to make educated choices about which websites to visit to research a topic. This helps reinforce optimal information seeking and use strategies, such as the need to read the content of Web pages carefully and critically.

Additionally, youth would benefit from instruction on bookmarking pages (so they don’t have to rely solely on their memories) and on distinguishing ads from true search results on search engine result pages. In acknowledgment of the former, we emphasize the need to record sources used in our pods on note-taking (discussed below). We also include instruction on how to use online bookmarking and organization services, such as Evernote.

Along with increasing their familiarity with health terminology, we need to ensure that they are aware of the limitations of relying solely on word familiarity when selecting search results. Youth would benefit from instruction on the difference between the words “useful” and “credible,” with special attention paid to pointing out that the presence of particular types of content (eg, pictures, videos) and/or particular types of functionality (eg, translation, read-aloud) is not, in and of itself, an indicator of credibility. Our pods now include examples of incorrect information that gets spread rapidly around the Internet, even being reposted or tweeted from otherwise reputable sources.

Regarding information assessment, we can help students by increasing their pre-existing knowledge and by pointing out that relying on one’s pre-existing knowledge when selecting a search result and when assessing the credibility of a site may prove unsuccessful if they have an insufficient or incorrect understanding of the topic they’re searching. Helping youth know where to look when trying to identify the author and the date of a site (or information on a site) is also extremely important, as is emphasizing the need to not only identify the name of the author, but also his or her qualifications. Nuanced instruction regarding the definitions of the different domain types and the general strengths and weaknesses of each of these types of sites is also called for. Helping students to develop better heuristics and engage in more conscious and effortful strategies in assessing the credibility of online information is also important. Included in our pods is an activity where students work in groups to develop their own heuristics. Increasingly important is imparting an understanding of the more social types of credibility measures, such as number of followers, number of viewers, and user ratings. Additionally, students need to understand the importance of consulting multiple sites to gather, cross-verify, and synthesize information, as well as the need to assess the credibility of each individual site even when in the process of conducting cross-verification, as this will help to improve their likelihood of ending up with truly credible information.

Moving to the latter phases of information management and use, instruction on note-taking is vitally important. Students need to know how to identify the relevant portions of information on a Web page and how to take good notes on this content, using their own words. Further, they need to understand what plagiarism is and why it is important to avoid. To address this need, we included activities focused on note-taking skills, plagiarism, and on the ethical use of information in our pods.

### Limitations

Our work blends research with practice—our research informs our practice, just as our practice informs our research. While this arrangement affords us with some important strengths, such as increased relevance and suitability of our instruction and research methods for our particular population, it also means that our findings may not be generalizable beyond the 30 youth who have participated in HackHealth so far. However, as we work with more and more youth across time, we will be able to ascertain whether there are particular patterns that tend to recur across both individuals and schools, which could suggest broader applicability of some of our methods and findings.

Another limitation of our work pertains to our use of the school library setting and of imposed tasks throughout the program. Although the use of a non-home setting for the program and the assignment of set tasks could lead to nonnatural behaviors, we attempted to limit the potential impacts of these decisions in three ways. First, we elected to use each group’s own school library setting, as it was already a part of the participants’ daily lives. We felt that this would increase their comfort level as they participated in the program and help to elicit more natural behaviors than an outside space might have done. Second, although we use set, imposed tasks, we nearly always do so in the context of health topics that the participants selected. In this way, we aimed to ensure each participant’s interest in his/her topic and the personal relevance of that topic for him/her. Third, we decided to use multiple data collection methods, so that we could analyze participants’ behavior from a variety of perspectives.

### Conclusions

Today’s youth have an unprecedented opportunity to live long and healthy lives; however, they need to have self-efficacy, information access, and a wide array of health literacy skills to do so. Our HackHealth after-school program for middle school students from socioeconomically disadvantaged backgrounds aims to capitalize on this population’s interest in science and health, simultaneously increasing their health-related motivation and self-efficacy, their digital and health literacy skills, and their understanding of the crucial link between their daily health-related behaviors and their ability to maintain their health and prevent disease. With an increased societal focus on health and a shift from viewing patients as passive recipients of medical care to viewing them as active arbiters of their own health, it will only become more imperative that youth possess these many different types of health literacy skills.

Although the vast majority of existing literature on health literacy assessment has focused on adults and has generally sought to measure reading comprehension, shifting our focus to this younger, more vulnerable (in terms of both age and socioeconomic class) population and widening our focus to encompass the much broader range of skills that actually constitute health literacy provides us with an opportunity to intervene at an early point in the pernicious cycle between poor health literacy and poor health outcomes. We can thereby contribute toward improving the long-term health outlook for this population and reducing health disparities. Furthermore, our intervention takes place at a critical stage in these individuals’ development, as adolescence is often the time when people begin to develop health-related habits and to enact (or not) particular health behaviors of their own accord. Moreover, this age range is very often the time when parents of children with chronic health conditions pass along self-care responsibilities, resulting in poorer health outcomes for those adolescents who fail to successfully adapt to this transition and undertake the necessary self-care activities on their own. By assessing and improving the health literacy skills of this population, we can increase their motivation and their belief in their ability to exert control over their own health, as well as their ability to find, understand, manage, and make use of credible health-related information.

## Abbreviations

DBR: design-based research
NAAL: National Assessment of Adult Literacy
REALM-Teen: Rapid Estimate of Adult Literacy in Medicine-Teen
